# Heat Stress Decreases Rice Grain Weight: Evidence and Physiological Mechanisms of Heat Effects Prior to Flowering

**DOI:** 10.3390/ijms231810922

**Published:** 2022-09-18

**Authors:** Chao Wu, Kehui Cui, Shah Fahad

**Affiliations:** 1 Guangxi Key Laboratory of Plant Functional Phytochemicals and Sustainable Utilization, Guangxi Institute of Botany, Guangxi Zhuang Autonomous Region and Chinese Academy of Sciences, Guilin 541006, China; 2 National Key Laboratory of Crop Genetic Improvement, MOA Key Laboratory of Crop Ecophysiology and Farming Systems in the Middle Reaches of the Yangtze River, Huazhong Agricultural University, Wuhan 430070, China; 3 Hainan Key Laboratory for Sustainable Utilization of Tropical Bioresource, College of Tropical Crops, Hainan University, Haikou 570228, China

**Keywords:** rice, heat stress, grain weight, panicle initiation, phytohormone

## Abstract

Heat stress during the preflowering panicle initiation stage seriously decreases rice grain weight in an invisible way and has not been given enough attention. The current review aims to (i) specify the heat effects on rice grain weight during the panicle initiation stage compared with the most important grain-filling stage; and (ii) discuss the physiological mechanisms of the decreased rice grain weight induced by heat during panicle initiation in terms of assimilate supply and phytohormone regulation, which are key physiological processes directly regulating rice grain weight. We emphasize that the effect of heat during the panicle initiation stage on rice grain weight is more serious than that during the grain-filling stage. Heat stress during the panicle initiation stage induces alterations in endogenous phytohormones, leading to the inhibition of the photosynthesis of functional leaves (source) and the formation of vascular bundles (flow), thus reducing the accumulation and transport of nonstructural carbohydrates and the growth of lemmata and paleae. The disruptions in the “flow” and restrictions in the preanthesis “source” tissue reduce grain size directly and decrease grain plumpness indirectly, resulting in a reduction in the final grain weight, which could be the direct physiological causes of the lower rice grain weight induced by heat during the panicle initiation stage. We highlight the seriousness of preflowering heat stress on rice grain weight, which can be regarded as an invisible disaster. The physiological mechanisms underlying the lower grain weight induced by heat during panicle initiation show a certain novelty because they distinguish this stage from the grain-filling stage. Additionally, a number of genes that control grain size through phytohormones have been summarized, but their functions have not yet been fully tested under heat conditions, except for the *Grain Size and Abiotic stress tolerance 1* (*GSA1*) and *BRASSINOSTEROID INSENSITIVE1* (*OsBRI1*) genes, which are reported to respond rapidly to heat stress. The mechanisms of reduced rice grain weight induced by heat during the panicle initiation stage should be studied in more depth in terms of molecular pathways.

## 1. Introduction

Global warming has raised the Earth’s average surface temperature, and extreme heat events have become more frequent worldwide [1]. On a regional scale, significant warming trends are observed in China (0.4 °C/decade), India (0.45 °C/decade), Spain (0.5 °C/decade), and Iran (0.6 °C/decade) [2,3,4,5]. As one of the most important staple crops, rice has experienced severe yield losses due to frequent heat events [6,7]. Recently, the occurrence of heat events has shifted to an earlier date. For example, heat events have been occurring in the rice growing regions of the Yangtze River basin in China in mid-July or even earlier [8]. In most rice paddy regions in China, the panicle initiation stage of middle rice often occurs in July, which begins when the panicle primordia have differentiated (approximately 30 days before heading) and ends when the pollen is fully matured [9]. The optimum and maximum temperatures for panicle initiation in rice are 26.7 °C and 33.1 °C, respectively [10]. However, the average maximum temperature in July in most rice growing regions in the Yangtze River basin has frequently surpassed 35 °C in recent years. The significance of heat stress during the preflowering periods in rice plants [11], especially the heat effects on meristem initiation and development of rice [12], has attracted attention and is emphasized as a key area for understanding plant heat stress responses in future studies.

Heat stress, which appears as an increase in temperature beyond a critical threshold (33.1 °C for panicle initiation) for a certain duration [10], occurs during the panicle initiation stage and leads to marked declines in the number of spikelets per panicle, spikelet fertility, and grain weight [13]. The effects of heat stress during the reproductive stage of rice on yield components as well as the underlying mechanism have been studied in depth [14,15]. However, studies of the heat effect on grain weight have focused mostly on the middle and late reproductive growth stages, i.e., the flowering to grain-filling stage [16], while the influence of heat stress on the grain weight of rice during the early reproductive growth stage, i.e., the panicle initiation stage, has not attracted much attention and is thus only superficially understood [6]. Rice grain weight depends on grain size (width, length, grain thickness) and grain plumpness (degree of filling) [17], both of which require assimilates as basic materials that are regulated by phytohormones [18]. In the current review, the effects of heat stress during the panicle initiation stage on the grain weight of rice are discussed with respect to two components: grain size and grain plumpness. Furthermore, the physiological mechanism through which heat stress during the panicle initiation stage influences the grain weight of rice is analyzed in terms of assimilate supply and phytohormone regulation.

## 2. Heat Effects on Rice Grain Weight during the Panicle Initiation Stage

**Figure 1:**
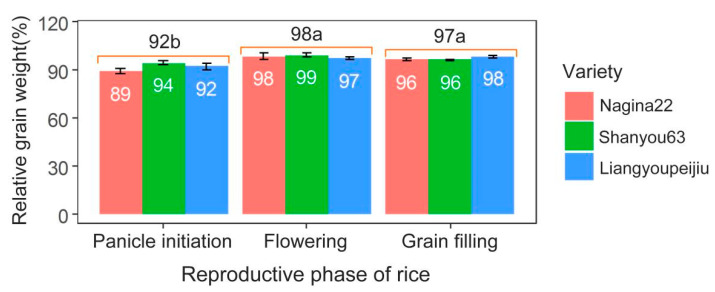
Effects of heat stress on rice grain weight during the panicle initiation, flowering, and grain-filling stages. Data originated from a location experiment repeated for 5 years [6] at Huazhong Agricultural University, Wuhan, China (30°29′ N, 114°22′ E). Heat treatments were imposed at panicle initiation, flowering, and grain-filling for 15, 7, and 30 days, respectively.

The grain weight of rice is severely decreased by heat stress during the panicle initiation stage, during which the heat effects on grain weight are even more severe than those in the grain-filling stage, according to Aghamolki et al. (2014) [19] and the observations of our 5-year case studies (Figure 1). Similarly, high soil temperature treatment significantly reduces the grain weight of rice during the panicle initiation stage but has a small or null effect on rice grain weight during the grain-filling stage [20]. It is estimated that the grain weight of rice is decreased by an average of 11.7% (5.4–17.1%) by heat during the panicle initiation stage [13,21,22,23]. Thus, the panicle initiation stage is one of the critical periods for rice grain weight under heat conditions, in addition to the grain-filling stage.

Grain size, which is established during the panicle initiation stage (the first critical stage), determines the maximum potential grain weight, and grain plumpness, which is established during the grain-filling stage (the second critical stage), determines the final grain weight [24]. Previous studies on the effects of heat stress on grain weight have mostly concentrated on the grain-filling stage, during which heat stress reduced grain weight by decreasing grain plumpness but not through grain size, and the reduced grain plumpness was mainly attributed to the affected physiological and biochemical processes such as sucrose unloading, conversion, and starch synthesis under heat stress during the grain-filling stage [25]. It is concluded that heat stress during the panicle initiation stage has serious adverse effects on rice grain weight, but the reasons for the effects of heat stress during the panicle initiation stage on the grain formation of rice are somewhat different from those during the grain-filling stage [18,26].

## 3. Heat-Induced Changes in Assimilate Supply Explain the Reduced Rice Grain Weight during the Panicle Initiation Stage

The reduced grain weight of rice induced by heat during the panicle initiation stage is associated with decreased grain size and grain plumpness, both of which require sufficient amounts of nonstructural carbohydrates (NSCs) as basic materials [27]. Heat stress inhibits the photosynthesis of functional leaves and thus reduces the preflowering production and accumulation of NSCs during the panicle initiation stage [28]. If combined with a high nighttime temperature, additional respiration-based carbohydrate consumption will be induced [29], resulting in further reduction of the preflowering accumulation of NSCs in the stems. Heat stress during the panicle initiation stage results in an inadequate accumulation and supply of NSCs [30] and further restricts the growth of lemmata and paleae [31], manifesting as a reduced grain size, which cannot be discerned until heading [13].

Heat stress during the panicle initiation stage reduces the number of vascular bundles and the area of the large and small vascular bundles in panicles, thus restricting the transport of NSCs toward the young panicles during the panicle initiation stage and hindering the transport of assimilates to the spikelets during the grain-filling period [32]. In fact, the panicle initiation stage is a critical period for the development of young panicles, the formation of vascular bundles, and the accumulation of NSCs in panicles prior to flowering under heat conditions [21,28,32,33]. As a result, insufficient and disrupted NSC accumulation prior to flowering restricts post-flowering grain-filling (endosperm proliferation and plumpness) and reduces grain plumpness, ultimately reducing rice grain weight.

**Figure 2:**
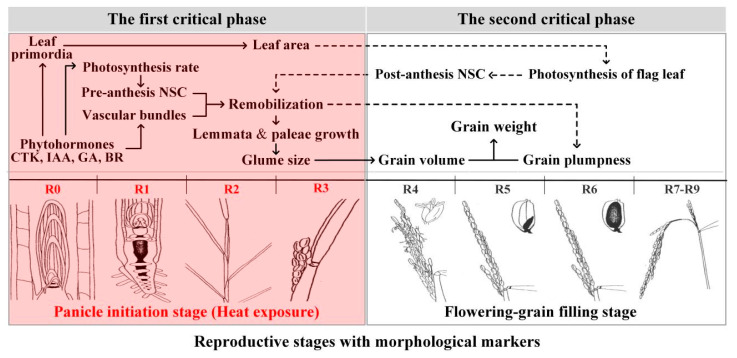
Proposed mechanisms of reduced rice grain weight in terms of phytohormonal regulation and assimilate supply under heat during the panicle initiation stage. R0: initiation of panicle development; R1: formation of panicle branches; R2: formation of the flag leaf collar; R3: panicle emergence from the boot; R4: at least one floret on the main panicle reach anthesis; R5–R6: one or more caryopsis on the main panicle elongate to the end of the hull; R7–R9: grains on the main stem panicle show yellow or brown hulls. (the illustrations of the reproductive stages with morphological markers are adapted from those by Wu et al. [18]).

Most studies on the influence of heat stress on grain weight have focused on the grain-filling stage. Rice glumes reach their final size at anthesis, and heat treatment during the grain-filling stage thus has no obvious influence on the length and width of rice grains [34,35]. However, heat stress during the panicle initiation stage directly reduces the length and width of grain [13] and indirectly reduces post-flowering grain plumpness, thereby resulting in decreased grain weight. Hence, the underlying mechanism through which heat stress induces a decrease in rice grain weight during the panicle initiation stage is different from that during the grain-filling stage. In summary, heat stress during the panicle initiation stage may simultaneously decrease both grain size and plumpness, which are associated with the assimilate supply (Figure 2). However, it is not clear exactly how heat stress reduces rice grain size and grain plumpness by regulating the supply of assimilates during the panicle initiation stage. The relationships between grain size, grain plumpness, and grain weight from the aspects of the assimilate supply and distribution are discussed as follows.

(i) Grain plumpness. The amount of NSCs in the stems decreased during the panicle initiation stage when plants were subjected to heat stress, but the heat-stressed plants showed a compensatory response after the heat stress was removed [36], which was reflected by a greater accumulation of aboveground biomass compared with that of the plants under control conditions. Deficit irrigation is usually adopted to ensure high and stable grain yields [37], which benefit mainly from the compensatory effect induced by regulating water availability [38]. However, how the compensatory effect induced by heat stress during the preflowering panicle initiation stage influences grain plumpness has rarely been evaluated. If heat stress during the panicle initiation stage exerts any effect on grain plumpness, is the outcome negative (zero or partial compensation) or positive (overcompensation)? If the effect is negative, what are the main reasons for the reduction in grain weight caused by heat stress? Furthermore, it is unclear whether the NSC/floret ratio (NSCs/number of florets) at heading decreases [39] and whether the quantity of NSCs increases due to the compensatory effect under heat conditions during the panicle initiation stage [36]. It is speculated that the amount of photosynthesis may not be the primary reason why heat stress restricts grain plumpness. Heat stress during the panicle initiation stage may affect grain plumpness by influencing the post-flowering remobilization of NSCs.

(ii) Grain size. To obtain heavy grain, breeding scientists usually aim to increase grain size, i.e., sink expansion, through genetic improvements [24], and cultivation experts generally advocate for “increasing the source and improving the flow”, in addition to “sink expansion”, to improve grain plumpness [40]. In fact, the variation in grain size resulting from cultural practices can cause a variation as high as 10% in grain weight [41]. In recent years, scholars have suggested increasing the grain weight by regulating the grain size using cultivation techniques [42]. Statistical analysis of existing data shows that heat events during the panicle initiation stage induce more than a 10% variation in grain weight [13,22,23], imposing a great impact on rice grain yield. Heat stress during the panicle initiation stage reduces the preflowering assimilate supply and thus decreases grain size, which restricts the sink capacity. Further studies should be performed to elucidate exactly how heat stress dominates rice grain weight during the panicle initiation stage.

## 4. Effect of Phytohormones on Grain Weight under Heat during the Panicle Initiation Stage

Rice grain weight is directly regulated by phytohormones such as cytokinins (CTKs), indoleacetic acid (IAA), gibberellic acids (GAs), and brassinosteroids (BRs) to a considerable degree [18,43,44,45]. IAA in the shoot apical meristem regulates leaf differentiation and further influences leaf area [46], CTKs affect the net photosynthetic rate of leaves [47] and aboveground biomass [48], and CTK, GA, and IAA regulate the development of vascular bundles in rice [49,50]. The physiological activities directly determine the preflowering accumulation and transport of NSCs and thus influence grain size and grain plumpness. IAA interacts with salicylic acid to mitigate injury during the differentiation and growth of spikelets under heat conditions [23], and the changes in endogenous CTK, IAA, and GA_1_ levels induced by heat are significantly correlated with heat-induced changes in grain weight during the panicle initiation stage [39]. Path analysis revealed that CTKs were most strongly correlated with grain weight (coefficient of determination of 0.91), followed by IAA (0.27), GA_1_ (0.25), and ABA (−0.08) (unpublished data). These results indicate that phytohormones, particularly CTKs, are strongly related to rice grain weight under heat stress during the panicle initiation stage.

Previous studies have shown that many genes or quantitative trait loci controlling rice grain weight also regulate grain size by influencing phytohormones (Table 1). For example, the *Cytokinin oxidase* (*OsCKX*), *PURINE PERMEASE* (*OsPUP*), MIKC^C^ class type II MADS-box gene (*OsMADS29*), and *DROUGHT AND SALT TOLERANCE* (*DST*) genes regulate grain size by influencing CTK contents. The *OsCKX* gene regulates the oxidative degradation of CTKs [51], the *DST* gene regulates CTK accumulation in the shoot apical meristem by interacting with *OsCKX2* directly [44], the *OsPUP4* gene regulates the long-distance transport of CTK [52], and the *OsMADS29* gene affects grain size and grain weight by regulating carbohydrate metabolism via CTKs [53]. The genes *GRAIN-LENGTH-ASSOCIATED* *2* (*GL2*), *GRAIN WIDTH* *5* (*GW5*), and *SLENDER GRAIN ON CHROMOSOME* (*SLG*) regulate grain size through BRs that possess antistress activity [54,55,56]. Among the genes regulating rice grain weight (Table 1), the *OsCKX2*, *DST*, a member of the CYP450 gene cluster (*DSS1*/*CYP96B*), *calcium-dependent protein kinase 1* (*OsCDPK1*), *Grain Size and Abiotic stress tolerance 1* (*GSA1*), and *BRASSINOSTEROID INSENSITIVE1* (*OsBRI1*) genes showed rapid and strong changes in their expression under abiotic stresses, such as heat, high light, salinity, and drought. However, the functions of the other genes in Table 1 have not yet been tested under abiotic stress and should be studied in more depth to clarify the molecular mechanisms for reducing rice grain weight under heat during the panicle initiation stage.

## 5. Conclusions and Perspective

Heat stress during the panicle initiation stage negatively affects grain in seemingly unapparent ways. The mechanism through which heat stress during the panicle initiation stage reduces grain size and grain plumpness is summarized in terms of assimilates and endogenous phytohormones. (i) Heat stress during the panicle initiation stage prevents the accumulation and sufficient supply of NSCs in young panicles, thus restricting spikelet growth and leading to reduced grain size. Moreover, the inadequate accumulation and impaired translocation of NSCs in the culm during the panicle initiation stage and the reduced sink size may restrict post-flowering grain plumpness. (ii) Heat stress during the panicle initiation stage influences the levels of endogenous phytohormones (CTKs, BRs, IAA, and GAs), thus affecting glume enlargement and the accumulation and transport of NSCs, further hindering preflowering glume growth and reducing post-flowering grain plumpness. There is no doubt that heat stress during the panicle initiation stage can reduce grain size and grain plumpness simultaneously, but it is still not clear exactly how heat stress dominates rice grain weight during the panicle initiation stage, which should be elucidated in further studies.

**Figure 3:**
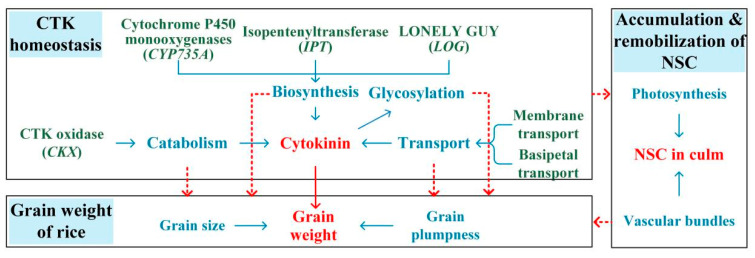
Proposed mechanisms underlying grain weight changes mediated by CTK under heat during the panicle initiation stage.

Phytohormone homeostasis (biosynthesis, catabolism, deactivation, and transport) is pivotal in regulating plant acclimation to environmental stress [18]. However, there is still a lack of comprehensive analysis and an overall understanding of the effects of heat stress on the processes involved in phytohormones homeostasis. The possible mechanisms of reduced rice grain weight in terms of CTK homeostasis under heat during the panicle initiation stage are proposed (Figure 3) based on the discussion above and our previous study [39]. Future studies on the physiological mechanisms of the reduced rice grain weight induced by heat stress during the panicle initiation stage should focus on the responses of processes involved in the homeostasis of other phytohormones, especially the anti-stress hormone BRs. Additionally, a number of genes have recently been identified to regulate grain weight through phytohormones (Table 1). However, most of the currently identified genes that play a role in grain weight are associated with grain size, but the genes that regulate grain plumpness have rarely been identified. The genes involved in grain size and grain plumpness should be further explored, and their functions should be given more attention under heat conditions during the panicle initiation stage.

Notably, the panicle initiation stage is the key stage for top-dressing fertilizers such as nitrogen, potassium, and phosphorus, which regulate source–sink relations and thus contribute to high grain yield [105]. Heat stress inhibited the remobilization of nitrogen, potassium, and phosphorus to panicles [106,107], and high levels of nitrogen and/or silicon application significantly impacted the aboveground biomass and rice grain weight under heat conditions during the panicle initiation stage [108,109]. In fact, fertilizer application usually interacts with heat treatments in terms of source–sink relations and yield formation [110]. Nitrogen and silicon may protect rice plants against heat injury because fertilizers delay senescence and enhance the synthesis of cytokinins [111,112], which tightly regulate source–sink relations and grain weight in rice plants (Figure 3). Furthermore, silicon fertilizer positively regulated the translocation efficiencies and allocation rates of nitrogen and potassium under heat conditions [107]. Thus, the effects of fertilizers on rice grain weight under heat stress during the panicle initiation stage should also be emphasized in further studies.

## Figures and Tables

**Table 1:** The genes controlling grain size.

Genes	Regulated Trait of Grain Size	Regulated Phytohormones	Verified Stresses	References
*RGB1*	Grain length	CTK	—	[57]
*OsER1*	Grain length	CTK	—	[58]
*OsPUP1*	Grain length, grain width	CTK	—	[59]
*AGO2*	Grain length	CTK	—	[60]
*OsCKX2*	Grain length, grain width	CTK	Salinity, drought	[51,61]
*OsSGL*	Grain length	CTK	—	[62,63]
*OsPUP4*	Grain width, grain thickness, grain length	CTK	—	[52]
*GNP1/GA20ox1*	Grain length	CTK, GA	—	[64]
*OsMADS29*	Grain length	CTK	—	[53]
*OsPUP7*	Grain length, grain width	CTK	—	[65]
*DST*	Grain length, grain width	CTK	Salinity, drought	[44]
*GW6*	Grain length, grain width	GA	—	[66]
*SGD2*	Grain length, grain width	GA	—	[67]
*OsGASR9*	Grain length, grain width, grain thickness	GA	—	[68]
*DSS1/CYP96B*	Grain length, grain width	GA, ABA	Drought	[69]
*SGL*	Grain length, grain width	GA	—	[45]
*OsCDPK1*	Grain length, grain width	GA	Drought	[70]
*OsARF6/OsAUX3*	Grain length	IAA	—	[71]
*GSA1*	Grain length, grain width	IAA	Heat, salinity, drought	[72]
*TSG1/FIB*	Grain length, grain width	IAA	—	[73]
*OsSK41/OsGSK5*	Grain length, grain width	IAA	—	[74]
*DS1/OsEMF1*	Grain length, grain width	IAA, BR	—	[75]
*Gnp4/LAX2*	Grain length	IAA	—	[76]
*SMOS1/DLT*	Grain length	IAA, BR	—	[77,78]
*BG1*	Grain length, grain width	IAA	—	[79]
*CYP78A13*	Grain length, grain width, grain thickness	IAA	—	[80]
*TGW6*	Grain length	IAA	—	[43]
*NAL2/3*	Grain width	IAA	—	[81]
*OsPIN2*	Grain length, grain width	IAA	—	[82]
*DSS1/OsDWARF*	Grain length, grain width	BR	—	[83]
*POW1*	Grain width, grain thickness, grain length	BR	—	[84]
*ZmD11*	Grain length	BR	—	[85]
*OsBRI1*	Grain length, grain width	BR	Heat, high light	[86,87]
*OsGRAS19/D26*	Grain length, grain width	BR	—	[88]
*GSN1*	Grain length	BR	—	[89]
*OsRac1*	Grain length, grain width	BR	—	[90]
*LTBSG1/BRD2*	Grain length, grain width	BR	—	[91]
*SRS5*	Grain length	BR	—	[92]
*GW5*	Grain width	BR	—	[56]
*DSG1*	Grain length, grain width	BR	—	[93]
*OFP1*	Grain width	BR	—	[94]
*DG1*	Grain length, grain width	BR	—	[95]
*CYP734A4*	Grain length	BR	—	[96]
*SMG11/D2*	Grain length, grain width	BR	—	[97]
*SLG*	Grain length, grain width	BR	—	[55]
*LHDD10/BRD2*	Grain length, grain width	BR	—	[98]
*CPB1/D11*	Grain length, grain width	BR	—	[99]
*GL2*	Grain length, grain width	BR	—	[54]
*OsMAPK6*	Grain length	BR	—	[100]
*sg4/D11*	Grain length	BR	—	[101]
*GS5*	Grain width	BR	—	[102]
*OsMKK4/SMG1*	Grain length, grain width	BR	—	[103]
*SRS3*	Grain length, grain width	BR	—	[104]

—: the gene function has not yet been tested under abiotic stress.

## Data Availability

All data are available in the manuscript.
